# Neutrophil Engulfment in Cancer: Friend or Foe?

**DOI:** 10.3390/cancers17030384

**Published:** 2025-01-24

**Authors:** Tong Lu, Wei Li

**Affiliations:** 1 Division of Hematology and Oncology, Department of Pediatrics, Penn State College of Medicine, Hershey, PA 17033, USA; 2 Penn State Cancer Institute, Penn State College of Medicine, Hershey, PA 17033, USA; 3 Department of Biochemistry and Molecular Biology, Penn State College of Medicine, Hershey, PA 17033, USA

**Keywords:** tumor-associated neutrophils, neutrophil engulfment, emperipolesis, phagocytosis, LC3-associated phagocytosis, tumor microenvironment, neutrophil-based drug delivery

## Abstract

Neutrophils, the most abundant circulating white blood cells, are essential for the initial immune response to infection and injury. Emerging research reveals a dualistic function of neutrophils in cancer, where they can promote or inhibit tumor progression. This dichotomy is influenced by the tumor microenvironment, with neutrophils capable of remodeling the extracellular matrix, promoting angiogenesis, or alternatively inducing cancer cell death and enhancing immune responses. An intriguing yet poorly understood aspect of neutrophil–cancer interactions is the phenomenon of neutrophil engulfment by cancer cells, which has been observed across various cancers. This process, potentially mediated by LC3-associated phagocytosis (LAP), raises questions about whether it serves as a mechanism for immune evasion or contributes to tumor cell death through pathways like ferroptosis. This review examines current knowledge on neutrophil development, their roles in cancer, and the mechanisms of LAP in neutrophil engulfment by tumor cells. We discuss how manipulating LAP impacts cancer progression and may represent a therapeutic strategy. We also explore neutrophils’ potential as delivery vehicles for cancer therapeutic agents. Understanding the complex functions of tumor-associated neutrophils (TANs) and the molecular mechanisms underlying LAP in cancer may open new avenues for effective therapeutic interventions and mitigate potential risks.

## Simple Summary

Neutrophils, the most abundant white blood cells crucial for fighting infections, exhibit both tumor-promoting and tumor-inhibiting roles in cancer. This dual behavior depends on the environment around the tumor and includes remodeling the extracellular matrix, fostering angiogenesis, inducing cancer cell death, and enhancing immune responses. A notable yet underexplored phenomenon is the engulfment of neutrophils by cancer cells, potentially mediated by LC3-associated phagocytosis (LAP). This process raises critical questions about its role in either aiding immune evasion or triggering cancer cell death through mechanisms such as ferroptosis. This review delves into neutrophil biology, their complex roles in cancer, the impact of LAP on neutrophil engulfment, and the therapeutic implications of modulating this pathway. Additionally, it highlights the potential of neutrophils as delivery systems for cancer therapies, underscoring the need to unravel tumor-associated neutrophil functions and LAP mechanisms to inform novel and effective cancer treatments.

## 1. Introduction

Neutrophils are the most abundant white blood cells in human circulation and account for 50–70% of the total white blood cell population [1]. They serve as one of the initial lines of defense against infection and injury through their traditional roles associated with acute inflammation, phagocytosing pathogens, and releasing cytotoxic granules to neutralize threats [2]. Emerging evidence reveals a more complex role of neutrophils in the tumor microenvironment (TME). Similarly to other immune cells, neutrophils exist in a heterogeneous population consisting of various subsets [3,4]. Tumor-associated neutrophils (TANs) may exist in anti-tumorigenic (N1) and pro-tumorigenic (N2) states [5]. TANs can support tumor progression by releasing matrix metalloproteinases (MMPs) to degrade the extracellular matrix (ECM), secreting vascular endothelial growth factor (VEGF) to upregulate angiogenesis, and releasing immunosuppressive cytokines to suppress the functions of other immune cells in the TME [6,7,8]. Conversely, they can also mount an anti-tumor response by inducing tumor cell death and enhancing the recruitment of other immune cells to the tumor site. This duality of neutrophil behavior suggests that their function in cancer is highly context-dependent, influenced by the tumor microenvironment and cancer type [1,9,10,11].

One intriguing yet less understood aspect of neutrophil–cancer interaction is the phenomenon of neutrophil engulfment by cancer cells. The observation of neutrophil-in-cancer cells in many types of cancers, including pleomorphic xanthoastrocytoma, along with lung, gall bladder, micropapillary, and gastric carcinomas [12,13,14,15,16,17], has raised questions about its role in cancer progression. Is neutrophil engulfment a mechanism by which cancer cells protect themselves from immune detection, or could this process lead to cancer cell death through the transfer of cytotoxic granules and induction of oxidative stress? This “friend or foe” dichotomy presents a critical, unexplored avenue in cancer research with potentially significant therapeutic implications.

This review aims to discuss current knowledge surrounding the mechanisms and consequences of neutrophil engulfment by cancer cells. We will explore the dual nature of this interaction, discussing both its pro-tumor and anti-tumor effects and examining how this phenomenon may shift depending on the context of the tumor microenvironment. Additionally, we will highlight the potential for targeting neutrophil–cancer cell interactions in therapeutic strategies.

## 2. Neutrophils and Their Roles in Cancer

### 2.1. Neutrophil Development and Release

Neutrophils originate from granulocyte–monocyte progenitor (GMP) cells in the bone marrow [18,19]. Within the GMP population, there are distinct subpopulations, including stem cells, mitotic cells, and post-mitotic cells [18]. Some GMPs differentiate into promyelocytes and start to express CD66b [4,20]. Further differentiation is driven by the expression of transcription factors such as signal transducers and activators of transcription 3 (STAT3), and receptors such as granulocyte colony-stimulating factor receptor (G-CSFR) [21]. The production of granulocyte colony-stimulating factor (G-CSF), crucial for neutrophil maturation, is regulated by lymphocytes through IL-17 signaling and by phagocytes through IL-23 signaling [22,23,24]. As promyelocytes continue to mature, they start to express CD11b and CD16, progressing through stages as myelocytes, metamyelocytes, banded neutrophils, and ultimately mature neutrophils [4].

Interestingly, the majority of mature neutrophils remain in the bone marrow, with only 1–2% of the total neutrophil population circulating in the bloodstream [25]. This retention in the bone marrow is primarily regulated by C-X-C motif chemokine 12 (CXCL12) and its receptor, C-X-C chemokine receptor type 4 (CXCR4) [26,27,28,29,30]. Resident osteoblast and stromal cells in the bone marrow produce CXCL12, which binds to CXCR4 on neutrophils, preventing their release into circulation. To induce neutrophil mobilization into the bloodstream, endothelial cells outside the bone marrow produce chemokines, such as CXCL1, CXCL2, and CXCL5, to attract neutrophils expressing CXCR2 [27,29,30,31,32]. Additionally, G-CSF can decrease the interaction between CXCR4 and CXCL12 to reduce neutrophil retention in the bone marrow [18]. G-CSF can also stimulate megakaryocytes to release chemokines that attract CXCR2-expressing neutrophils, further facilitating their emigration into circulation [31].

### 2.2. Neutrophil Functions in Innate Immunity

Neutrophils serve as an integral part of the innate immune system to combat infections. In order to reach the inflamed tissue, mature neutrophils must go through sequential steps, including an adhesion cascade consisting of initial attachment to the surrounding endothelium of the blood vessel, followed by rolling along the endothelium, arresting with neutrophil spread, crawling, and finally the transmigration of the neutrophil through the basement membrane into the tissue of interest [33]. Attachment and rolling are mediated by integrins and P-, E-, and L-selectins [33]. As neutrophils roll, they undergo arrest when encountering neutrophil-recruiting chemokines such as CXCL6, CXCL8, and CCL3 [34,35]. The arrest step is mediated by neutrophil β_1_ and β_2_ integrins interacting with intercellular adhesion molecules (ICAM-1 and ICAM-2) expressed on inflamed endothelial cells [33]. During the transmigration step, neutrophils can migrate through the endothelium layer by going in between endothelial cells (paracellular migration) or through endothelial cells (transcellular migration). The transmigration step is mediated by various adhesion molecules, including ICAM-1, vascular cell adhesion molecule 1 (VCAM-1), platelet endothelial cell adhesion molecule 1 (PECAM-1), and cluster of differentiation 99 (CD99) [36,37]. Neutrophil lymphocyte function-associated antigen 1 (LFA-1) is the main integrin that interacts with endothelial ICAM-1 to mediate transmigration [38].

After entering the inflammatory tissue site, neutrophils can be primed and activated by pro-inflammatory stimuli, which include lipopolysaccharides, pro-inflammatory cytokines, and growth factors [39,40,41]. Neutrophils may also be activated by pathogen-associated molecular patterns (PAMPs) found on microbes and damage-associated molecular patterns (DAMPs) found on necrotic cells [33]. Upon activation, neutrophils can utilize various mechanisms to kill pathogens. First, neutrophils can directly phagocytose microbes in a mechanism mediated by Fcγ receptors and C-type lectin receptors [42,43]. The engulfed microbe is encapsulated in a vacuole, which fuses with neutrophil granules containing hydrolytic enzymes such as NADPH oxidase, myeloperoxidase (MPO), elastase, and proteinase 3 to form a mature phagosome [33]. The engulfed microbe is rapidly killed as the phagosome matures. One of the main ways neutrophils kill pathogens is through reactive oxygen species (ROS). Neutrophils primarily produce ROS through two main mechanisms. First, the NADPH oxidase complex, composed of four cytosolic subunits (RAC1, p40^phox^, p47^phox^, and p67^phox^) and two membrane-bound subunits (p22^phox^ and gp91^phox^ (NOX2)), generates ROS inside the phagosome lumen [44]. Additionally, MPO can also catalyze the generation of hypochlorous acid from hydrogen peroxide and chloride [45]. In addition to the fusion of enzyme-containing granules to the phagosome, neutrophils may also secrete these granules to the extracellular space for anti-microbial functions [33].

Additionally, neutrophils can use MMPs for anti-microbial functions [46]. Neutrophils produce inactive precursor versions of MMP-8 and MMP-9, known as pro-MMP-8 and pro-MMP-9, respectively [47]. Upon activation, neutrophils produce the active forms of the MMPs (MMP-8 and MMP-9) and release them into the extracellular space to help kill pathogens [46]. MMP-8 and MMP-9 can also degrade components of the extracellular matrix, which can promote immune cell infiltration into the inflammatory tissue [48].

Lastly, neutrophils can also use NETosis to kill pathogens. NETosis is characterized by the release of decondensed chromatin and granules from neutrophils [33]. The process requires NADPH oxidase. It is thought that NADPH oxidase-generated ROS may be necessary for the inactivation of caspases to inhibit apoptosis and allow for membrane lysis to occur [49]. Moreover, histone citrullination catalyzed by peptidyl arginine deiminase 4 (PAD4) is necessary for chromatin decondensation and maximal neutrophil extracellular trap (NET) dispersion [50]. During NETosis, neutrophils lyse and release chromatin and NETs into the extracellular space to trap and kill pathogens [33].

### 2.3. Neutrophil Functions in Cancer

#### 2.3.1. Pro-Tumor Roles of Neutrophils

First, neutrophils have been implicated in promoting tumorigenesis and cancer progression through inducing DNA damage. The ROS produced by neutrophils can induce DNA damage, potentially increasing the mutational burden in healthy cells and thereby contributing to tumorigenesis [51]. For instance, neutrophils have been shown to increase the levels of 7-hydro-8-oxo-2′deoxyguanosine (8-oxodG), a marker of ROS-induced DNA damage, in rat alveolar epithelial cells [52]. Additionally, myeloid cells produce ROS that can initiate tumorigenesis by inducing oxidative DNA damage in intestinal epithelial cells [53]. Activated neutrophils have been reported to release microparticles containing pro-inflammatory microRNAs, such as miR-23a and miR-155. These microRNAs promote lamin B1-dependent replication fork collapse and inhibit homologous recombination, resulting in double-strand DNA breaks [54].

Second, neutrophils can promote cancer progression by releasing granules containing various enzymes. One such enzyme, matrix metalloproteinase 9 (MMP-9), facilitates extracellular matrix remodeling and promotes angiogenesis to support tumor growth [55]. Deryugina et al. reported that the infiltration of MMP-9-secreting TANs into the TME correlates with increased angiogenesis and metastasis in various in vivo cancer models [56]. This effect may be explained by the ability of MMP-9 to degrade the ECM, which upregulates angiogenesis via the release of VEGF and promotes intratumoral neovascularization [57]. Neutrophils also release elastase, which degrades insulin receptor substrate-1 (IRS-1), enhancing tumor cell proliferation in a K-RAS-driven lung cancer mouse model [58]. The neutrophil elastase-mediated degradation of IRS-1 promoted interaction between phosphatidylinositol 3-kinase (PI3K) and platelet-derived growth factor receptor (PDGFR), which upregulated cancer cell proliferation and survival [58]. Moreover, neutrophils’ release of NETs has been reported to promote metastasis in 4T1 breast cancer cells and proliferation in anaplastic thyroid cancer cells [59,60]. Interestingly, the presence of NETs correlates with metastasis in breast cancer models [59]. Conversely, inhibiting NET formation and release by targeting NADPH oxidase, G-CSF, cathepsin G, and elastase decreased invasion in breast cancer cells [59]. Further investigation showed that using DNase I-coated nanoparticles to degrade NETs can reduce the lung metastasis of 4T1 cells in mice [59].

Furthermore, neutrophils can contribute to an immunosuppressive tumor microenvironment by modulating the functions of other immune cells. Studies have reported that neutrophils can suppress cytotoxic T cell activity through the release of arginase-1 and ROS, thereby inhibiting the immune response against tumors [61,62]. Arginase-1 released by neutrophils into the TME degrades extracellular arginine, which is needed for T cell proliferation, survival, activation, and anti-tumor functions [61,63]. Additionally, ROS released by neutrophils can suppress T cell function by impairing T cell receptor (TCR) signaling [62]. Neutrophil-derived ROS can damage T cells and suppress T cell proliferation and activation [64]. Moreover, Malmberg et al. reported that ROS can inhibit T cell function by blocking NF-κB activation [65]. Next, neutrophils can express immune checkpoint inhibitory receptors such as programmed death-ligand 1 (PD-L1) to suppress T cell activity and proliferation in the TME [66,67]. TANs have also been shown to secrete CCL17, which attracts regulatory T cells (T-regs) to the tumor site and promotes an immunosuppressive environment that supports tumor growth [68]. Furthermore, the phagocytosis of apoptotic neutrophils by macrophages can induce a shift in macrophages toward an immunosuppressive M2 state [69,70,71]. Altogether, these mechanisms allow neutrophils to promote tumorigenesis and cancer progression.

#### 2.3.2. Anti-Tumor Roles of Neutrophils

Neutrophils exhibit anti-tumor functions by inducing tumor cell death through various mechanisms. These include the release of nitric oxide [72] and the expression of Fas ligands (FasL), which induce apoptosis and cell cycle arrest in cancer cells [73]. Neutrophils also facilitate antibody-dependent cell-mediated cytotoxicity (ADCC) [74,75,76,77,78], and release MPO and H_2_O_2_ [79,80,81] to induce cancer cell death. Additionally, neutrophil-derived elastase can kill cancer cells by directly cleaving Fas receptors (CD95) on cancer cells, triggering apoptosis [82]. Furthermore, neutrophils can express tumor necrosis factor-related apoptosis-inducing ligand (TRAIL), which induces apoptosis in cancer cells [83].

Neutrophils can also activate other immune cell anti-tumor functions. For example, activated neutrophils secrete IL-18 and various natural killer (NK) cell-activating ligands to recruit and activate NK cells to suppress tumor growth [84]. In addition, neutrophils can cooperate with T cells for anti-tumor activities. Interestingly, treating primary tumors with neutrophil-derived elastase can attenuate tumor growth at secondary sites in a CD8^+^ T cell-mediated manner in triple-negative breast cancer and melanoma mice models [82]. The exact underlying mechanisms that mediate this abscopal effect remain to be elucidated. Additionally, activated T cells release interferon-gamma (IFN-γ) to induce pro-inflammatory interferon response transcription factor 1 (IRF-1) signaling in neutrophils [85]. In a positive-feedback loop, these activated neutrophils then induce macrophages to release interleukin 12 (IL-12), which activates more T cells and, in turn, causes them to produce IFN-γ [86]. Neutrophils can also use NETs to prime T cells and lower their activation threshold, enhancing the immune response against tumors [87]. Furthermore, an intriguing hybrid variant of neutrophils has been reported that can act as antigen-presenting cells to stimulate CD8^+^ T cell activity and contribute to anti-tumor immunity [88,89,90]. These hybrid neutrophils capture and process tumor antigens and present them to T cells via major histocompatibility complex (MHC) molecules to induce T cell proliferation and cytokine production [88,89,90].

Why neutrophils can play both pro- (N2) and anti-tumor (N1) roles is an intriguing and unresolved question. It was suggested that the opposite impacts on tumors by neutrophils could be determined by the specific context of the TME, in which TGF-β can drive neutrophil N2 polarization, whereas IFN-β leads to an N1 phenotype [91]. This N1 and N2 polarization appears to be associated with the tumor developmental stages. Friedmann-Morvinski and colleagues showed in a mouse glioblastoma model that the depletion of neutrophils at the tumor initiation stages can promote tumor growth, suggesting an N1 phenotype. However, neutrophils isolated at later tumor progression stages display pro-tumor properties [92]. Therefore, the TME factors driving N1 or N2 polarization and tumor developmental stages need to be considered when examining and targeting neutrophils in cancer.

### 2.4. Neutrophils as a Vehicle for Cancer Therapy

As key players in the immune response, neutrophils are capable of migrating across the endothelial barrier, navigating complex TMEs, and interacting closely with tumor cells. The migration is achieved through hijacking similar mechanisms used when neutrophils infiltrate the inflammatory tissue site during the innate immune response as described above (see Section 2.2). These characteristics make them an attractive tool for targeted drug delivery in anti-cancer strategies [93]. Several studies have demonstrated the potential of neutrophils for drug delivery in cancer therapy. For example, Zhang and colleagues showed that neutrophils loaded with paclitaxel-containing liposomes can migrate to the brain and suppress glioma relapse in mice after the initial tumor has been surgically removed [94]. The same research group later demonstrated that human neutrophils carrying Abraxane, an albumin-bound paclitaxel nanoparticle, can home to tumor sites in an ectopic gastric cancer model [95]. In both cases, an inflammatory treatment, e.g., surgical removal of primary tumors or radiation, was used to promote the homing of neutrophils to the tumor sites. Furthermore, Li and colleagues showed that neutrophils can be engineered to carry nanosensitizers that can be activated by light or ultrasound to generate ROS or release drug cargo. This approach has been used in inhibiting the growth of breast cancer and glioblastoma in preclinical mouse models [96,97]. Beyond conventional neutrophils, advances in cell engineering have led to the development of chimeric antigen receptor (CAR) neutrophils from human pluripotent stem cells. Bao and colleagues have used this approach and demonstrated that CAR-neutrophils can be loaded with nanoparticles and exhibit enhanced cytotoxicity against tumor cells in glioblastoma mouse models [98,99].

## 3. Neutrophil Engulfment by Non-Tumor Cells

In normal immune homeostasis, dead neutrophils are routinely engulfed by macrophages through a process known as efferocytosis. This process plays a key role in resolving inflammation [100]. In addition to the macrophage-mediated efferocytosis of neutrophils, other cell types have also been reported to engulf dead neutrophils. Quarato et al. reported that bone marrow mesenchymal stromal cells (MSCs) can also perform efferocytosis in end-stage neutrophils in the bone marrow microenvironment [101]. A murine bone marrow-derived stromal cell line, ST2, when engulfing end-stage neutrophils, had downregulated metabolic and biogenesis pathways, and upregulated cellular senescence and apoptosis pathways [101]. Additionally, the efferocytosis of end-stage neutrophils by ST2 cells impaired osteoblastic differentiation, oxidative phosphorylation, and glycolysis [101]. The molecular mechanisms of the phenomenon appear to be dependent on mitochondrial fission, as preventing mitochondrial fission decreases efferocytosis and rescues ST2 cells from differentiation impairments [101].

Live neutrophils can also be internalized by other cells. The process of live neutrophil engulfment by a host cell is termed emperipolesis, which refers to a phenomenon in which a live cell is “wandering about” inside another cell [102]. Cunin et al. reported that megakaryocytes are capable of engulfing live neutrophils [103,104]. After engulfment, neutrophils exit the engulfed vacuole and traverse through the megakaryocyte cytoplasm to reach the demarcation membrane system and donate neutrophil membrane components to the host megakaryocyte cell for platelet production [103,104]. Interestingly, the neutrophils then egress from the megakaryocytes intact [103,104]. Emperipolesis is more frequently found during inflammation in mice that exhibit platelet overproduction, suggesting it contributes to platelet production [103]. Further studies of this neutrophil-in-megakaryocyte phenomenon found that engulfed neutrophils go through at least two distinct processes which show different exiting times and morphologies, suggesting emperipolesis may have divergent functions [105]. Studies of neutrophil emperipolesis by megakaryocytes found an engulfment process dependent on intercellular adhesion molecules (ICAMs) and LFA-1, as well as ezrin [103,106]. Recently, Mihlan et al. reported that degranulating mast cells release leukotriene B4 to attract neutrophils. Once recruited, these live neutrophils are engulfed and digested by the mast cells. During this process, mast cells retain neutrophil-derived contents inside vesicles, which can be later released into the extracellular space for immune functions [107]. These secreted vesicles contain active neutrophil-derived contents such as MPO, enzyme-containing granules, and MMPs [107]. Additionally, the secreted neutrophil contents can also enhance pro-inflammatory functions in nearby macrophages by inducing type 1 interferon signaling [107]. Intriguingly, mast cells that engulf and digest neutrophils exhibited increased metabolic fitness and were more resistant to nutrient starvation [107]. Altogether, this neutrophil trapping phenomenon seems to boost mast cell immune functions and improve mast cell overall fitness [107].

## 4. Neutrophil Engulfment by Cancer Cells

### 4.1. Neutrophil Internalization by Cancer Cells

Engulfment involving neutrophils has also been observed in various cancers (Table 1), including pleomorphic xanthoastrocytoma, spindle cell squamous cell carcinoma, and buccal mucosa squamous cell carcinoma, as well as pancreatic, intestinal, lung, gall bladder, micropapillary, and gastric carcinomas [12,13,14,15,16,17,108,109,110,111]. However, its underlying mechanism and role in cancer progression remains to be investigated. Notably, emperipolesis can also refer to the engulfment of lymphocytes by host cells [102]. Burns et al. reported that the emperipolesis of lymphocytes by melanoma cells correlates with cancer cell death [112]. Additionally, Saxena et al. reported that breast cancer cells with lymphocyte emperipolesis have enhanced cytotoxic response to chemotherapy, potentially due to synergistic effects with lymphocyte-derived chemokines [113]. These studies suggest that lymphocyte emperipolesis by tumor cells may have anti-tumor effects.

### 4.2. LC3-Associated Phagocytosis Mediates Neutrophil Engulfment by Tumor Cells

Recently, Lu et al. demonstrated that glioblastoma cells are capable of engulfing neutrophils [118]. During this process, the internalized neutrophils are encircled by microtubule-associated protein 1A/1B-light chain 3 (LC3), resembling the similar phenomenon of LC3-associated phagocytosis (LAP) [119,120]. Once internalized, the neutrophils are fragmented, releasing their contents, including myeloperoxidase, within the tumor cells. This release induces tumor cell death through ferroptosis, which contributes to tumor necrosis during glioblastoma progression [81,118]. MPO, a peroxidase enzyme primarily found in neutrophils that catalyzes the generation of hypochlorous acid, can upregulate lipid peroxides in glioblastoma cells to induce ferroptosis, a form of non-apoptotic, regulated, iron-dependent cell death that is induced by lipid peroxidation-mediated membrane damage [81,118]. The apparent suicide of cancer cells following the engulfment of neutrophils requires key components of the LAP PI3KC3 complex, including vacuolar protein sorting 34 (VPS34), UV radiation resistance-associated gene protein (UVRAG), and RUN domain and cysteine-rich domain-containing Beclin-1-interacting protein (RUBCN) [118]. These findings suggest that LAP plays a critical role in the neutrophil engulfment process by cancer cells.

### 4.3. LC3-Associated Phagocytosis

**Figure 1:**
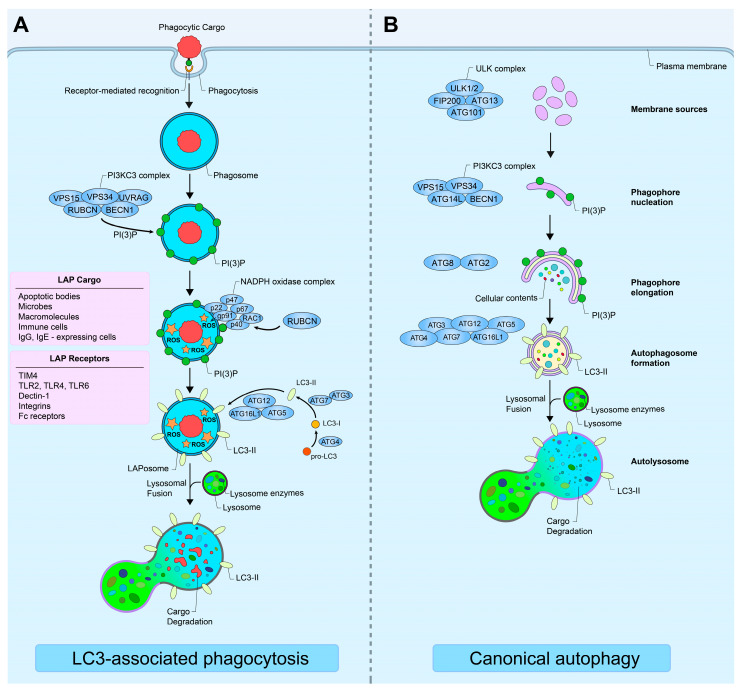
Mechanisms of LC3-associated phagocytosis and canonical autophagy. (**A**) Schematic diagram depicting LC3-associated phagocytosis mechanisms. Extracellular targets bind to receptors on the cellular plasma membrane to initiate phagosome formation. The cargo is then encapsulated inside a single-membrane phagosome and phagocytosed. The PI3KC3 complex, composed of VPS34, VPS15, BECN1, UVRAG, and RUBCN, is recruited to the phagosome and generates PI(3)P on the membrane. Next, the NADPH oxidase complex, consisting of p22, p47, gp91, p67, p40, and RAC1, generates ROS in the phagosome lumen. This NADPH oxidase complex is stabilized by RUBCN. Cytosolic pro-LC3 is converted into LC3-I by ATG4, and the LC3-I is then processed by ATG7-ATG3 and ATG12-ATG5-ATG16L1 complexes to form LC3-II on the phagosome membrane. The phagosome, now decorated with LC3-II and referred to as a LAPosome, fuses with lysosomes. Finally, lysosomal enzymes degrade the cargo in the LAPosome. (**B**) Schematic diagram depicting canonical autophagy mechanisms. Autophagy is induced by cellular signals such as nutrient deprivation and metabolic stress. During initiation, the ULK complex (composed of ULK1/2, ATG13, FIP200, and ATG101) and the PI3KC3 complex (composed of VPS15, VPS34, ATG14L, and BECN1) are activated. Membrane isolation occurs, and membrane components are derived from cellular sources such as the endoplasmic reticulum. The PI3KC3 complex generates PI(3)P on the early phagophore. Next, ATG8 and ATG2 mediate phagophore elongation around cellular contents designated for degradation. Various ATG proteins catalyze the lipidation of LC3-I to form LC3-II (see (**A**) for details), which are decorated on the double-membrane autophagosome. Eventually, the autophagosome fuses with lysosomes to form an autolysosome and the enclosed contents are degraded.

LAP combines elements of phagocytosis with components used in canonical autophagy [121,122,123] (Figure 1). Often regarded as a form of noncanonical autophagy, LAP allows phagocytic cells to internalize extracellular cargo and direct it to lysosomes for degradation [122,123]. This mechanism is commonly employed by the immune system for antimicrobial functions [124]. First, LAP is essential for the clearance of *Listeria monocytogenes* by macrophages in mice [125]. Additionally, macrophages utilize LAP to clear *Candida albicans* in a Dectin-1-dependent manner [126,127]. Dectin-1, a C-type lectin receptor expressed on the surface of phagocytic cells, is also required for the clearance of *Aspergillus fumigatus* [128]. In support of this, mice with impaired LAP function exhibit difficulty in clearing *Aspergillus fumigatus* infections [129]. In addition to its role in pathogen clearance, LAP may also regulate immune functions. For example, dendritic cells with impaired LAP function exhibit reduced ability to induce T-regs, which are important for suppressing inflammation [130].

LAP also plays a crucial role in clearing dead cells and apoptotic bodies through efferocytosis [120,131]. Phagocytic cells recognize “eat me” signals expressed on the surface of apoptotic cells. Subsequently, these dead cells are engulfed by phagocytes and processed for lysosomal degradation [120,131]. This lysosomal degradation not only removes dead cells, but also promotes the production of anti-inflammatory cytokines and the suppresses the production of pro-inflammatory cytokines [132,133,134,135,136,137]. The efferocytosis of apoptotic neutrophils by macrophages activates STAT3 signaling within macrophages, which causes macrophages to shift towards an immunosuppressive and anti-inflammatory M2 state [69,70,71,120,132]. This highly regulated process allows the immune system to efficiently clear dead cells, preventing chronic inflammation. Moreover, the lysosomal degradation of the internalized cargo not only clears cellular debris, but also facilitates the recycling of nutrients and triggers signaling cascades that regulate cellular functions [121,138].

Entosis, a non-apoptotic cell death process in which one cell is engulfed by another, also appears to depend on LAP [119,139,140]. Live MCF-7 breast cancer cells are internalized upon matrix detachment and nutrient deprivation by neighboring MCF-7 cells through a process dependent on Rho-ROCK signaling, actin, and myosin II [140,141]. Once engulfed, the internalized cell becomes enclosed in an entotic vacuole, which recruits LC3, a hallmark of LAP [139]. The knockdown of key LAP proteins, such as VPS34, autophagy-related 5 (ATG5), and autophagy-related 7 (ATG7), reduces the percentage of LC3-positive entotic vacuoles and diminishes the killing of internalized cells [139]. Eventually, the LC3-decorated entotic vacuole fuses with the lysosomes, leading to the degradation of the internalized cell [139]. Interestingly, subsequent studies have shown that not all internalized cells are killed; some may remain viable, proliferative, and even escape from the host cell [119].

### 4.4. Mechanisms of LC3-Associated Phagocytosis

LAP is initiated when a phagocytic cell recognizes extracellular targets in a receptor-dependent manner [121,122,123]. Various receptors have been implicated in the internalization of different targets, which can range from microbes and apoptotic cells to protein aggregates (Figure 1) [121,142]. Some known LAP receptors include toll-like receptors (TLRs), pattern recognition receptors, RGDS-motif-containing integrins, C-type lectin domain family 7 member A (Dectin-1) receptors, and the phosphatidylserine receptor T cell immunoglobulin and mucin domain containing 4 (TIM-4) [118,121,123,126,127,128,143,144,145]. Lu et al. reported that the engulfment of neutrophils by glioblastoma cells, as well as neutrophil-induced tumor cell death, can be blocked by RGDS peptides. Compared to naïve HL-60 human promyelocytic leukemia cells, which cannot be engulfed by tumor cells, differentiated HL-60 (dHL-60) neutrophils, which can be engulfed by tumor cells, express several integrin family genes, such as ICAM-1, ITGAM, ITGB2, ITGAX, and ITGAV, at higher levels [118]. The depletion of ITGAV from dHL-60 cells reduces the cell killing of tumor cells by dHL-60 cells, suggesting ITGAV is involved in mediating the interaction between the two cells [118]. Therefore, RGDS-motif-containing integrins could be engaged as receptors for the LAP of neutrophils by tumor cells [118].

Once a target is recognized, it is encapsulated in a single-membrane phagosome, which is then internalized. The class III phosphatidylinositol 3-kinase (PI3KC3) complex consisting of VPS34, phosphoinositide 3-kinase regulatory subunit 4 (VPS15), UVRAG, Beclin-1 (BECN1), and RUBCN is recruited to the phagosome, decorating it with phosphatidylinositol-3-phosphates (PI(3)P) [122,123,146]. Subsequently, the NADPH oxidase complex, composed of four cytosolic subunits (RAC1, p40^phox^, p47^phox^, and p67^phox^) and two membrane-bound subunits (p22^phox^ and gp91^phox^ (NOX2)), generates ROS within the phagosome lumen [134,147,148,149,150,151]. Next, cytosolic pro-LC3 is processed by ATG4 and converted into LC3-I, which is then lipidated by the ATG7-ATG3 and ATG12-ATG5-ATG16L1 complexes to form LC3-II on the phagosome membrane [122]. The LC3-II-decorated phagosome, now referred to as a LAPosome, fuses with lysosomes, where lysosomal enzymes ultimately degrade LAPosome cargo [122,144].

### 4.5. LC3-Associated Phagocytosis in Cancer

Several studies have investigated the impact of targeting LAP on cancer progression. Firstly, Cunha et al. reported that LAP-deficient myeloid cells led to a decrease in tumor growth in melanoma, Lewis lung carcinoma, and adenocarcinoma mouse models [152]. Interestingly, only the depletion of LAP-associated proteins (VPS34, RUBCN, ATG5, ATG16L, ATG7, and NOX2) suppressed the growth of B16 melanoma tumors, and this phenotype was not observed when targeting components involved in canonical autophagy, such as ATG14, ULK1, and FIP200 [152]. Additionally, tumor-associated macrophages (TAMs) with functional LAP exhibited an immunosuppressive state, whereas LAP-deficient TAMs displayed a pro-inflammatory phenotype with upregulated STING-dependent type I IFN activity, enhancing T cell effector functions in the tumor microenvironment [152]. Further supporting these findings, Noman et al. demonstrated that targeting VPS34 can suppress tumor growth and improve survival in various tumor models by increasing the infiltration of CD4^+^ T cells, CD8^+^ T cells, and NK cells into the tumor microenvironment [153]. VPS34 inhibition promoted the release of the pro-inflammatory chemokines CXCL10 and CCL5 via STAT1/IRF7 induction within tumors [153]. Intriguingly, the inhibition of VPS34 improved the efficacy of anti-PD-L1 immunotherapy in colorectal and melanoma cancer models [153]. Consistently, Lu et al. showed that the depletion of VPS34 in glioblastoma cells reduced tumor necrosis and increased survival in tumor-bearing mice with a glioblastoma mouse model. The effect was attributed to the inhibition of neutrophil-induced tumor cell ferroptosis by blocking LAP-mediated neutrophil engulfment by tumor cells [118]. Conversely, Moore et al. reported the anti-cancer effects of LAP in an acute myeloid leukemia (AML) model [154]. Bone marrow macrophages engulfed and processed AML-derived apoptotic bodies through LAP, which resulted in the induction of STING-dependent interferon responses that suppressed AML progression [154]. Furthermore, the activation of STING-dependent inflammatory pathways in TAMs was induced by the LAP-mediated processing of mitochondrial DNA from AML-derived apoptotic bodies [154].

## 5. Conclusions and Outlook

Accumulating evidence indicates that neutrophils significantly influence tumorigenesis and tumor progression. The anti-tumor and pro-tumor roles played by TANs appear to be shaped by the tumor microenvironment at various stages of tumor development. Understanding the multifaceted functions of TANs requires examining their interactions with tumor cells and other components of the tumor microenvironment. The engulfment of neutrophils by tumor cells is a phenomenon that has been repeatedly reported in various cancer types (Table 1) [12,13,14,15,16,17,102,108,109,110,111]. However, the functional implications and molecular mechanisms underlying the phagocytosis of immune cells by cancer cells remain largely unexplored.

Emerging evidence suggests that cancer cells may utilize LAP to engulf and process neutrophils, and inhibiting this process has been shown to reduce tumor necrosis and improve survival in a glioblastoma mouse model [118]. It would be beneficial to investigate whether targeting LAP could induce similar effects in other cancer types where neutrophil emperipolesis has been reported. Beyond their pathological roles, neutrophils have also been exploited as vehicles for delivering therapeutic agents in cancer treatment. The propensity of tumor cells to internalize neutrophils may enhance this application by improving delivery efficiency and minimizing off-target effects on healthy cells.

The core proteins of the LAP PI3KC3 complex (VPS34, VPS15, BECN1, UVRAG, RUBCN) present potential targets for developing novel cancer therapies. However, given that LAP is known to be involved in anti-inflammatory pathways such as the efferocytosis of apoptotic bodies and the anti-microbial clearance of bacterial and fungal infections, systematically targeting the LAP PI3KC3 complex could have undesirable side effects that result in chronic inflammation and prolonged infections. Additionally, targeting the LAP PI3KC3 complex may impair cellular vesicular trafficking, leading to the disruption of vital functions in healthy cells. One option to circumvent these potential side effects is the targeted delivery of therapeutic agents to reduce the impact on healthy cells. An alternative strategy could involve targeting specific upstream receptors that mediate the immune cell-to-cancer cell interaction that is required for LAP initiation. Integrin and its downstream signaling are deregulated and play important roles in cancer progression [155,156]. Given the potential role of integrin in mediating the neutrophil–cancer cell interaction [118], targeting the involved integrin may be plausible. In addition, TLRs frequently show elevated expression in various cancers [157]. Further investigation into these potential receptors is warranted to better understand the complex molecular mechanisms of this noncanonical form of autophagy in cancer in order to develop novel therapeutic approaches.

## Figures and Tables

**Table 1:** Neutrophil internalization by cancer cells.

Cancer Type	Observation Method	Cells Involved	Functional Implications	References
Various cancer cell types tested in vitro	Cell types were co-cultured in vitro followed by DAPI or H&E	HL-60 cells were internalized by A431 epidermoid carcinoma, MCF-7 breast cancer, MDA-MB-468 breast cancer, PLC/PRF/5 hepatoma, HCC-LM3 hepatocellular carcinoma, K562 leukemia, SK-BR-3 breast cancer, and BxPC-3 pancreatic adenocarcinoma cells.	Not studied	[114]
Gall bladder anaplastic carcinoma, small intestine adenocarcinoma, pancreatic adenocarcinoma, breast infiltrating duct adenocarcinoma, larynx squamous cell carcinoma, lung small cell carcinoma, anaplastic carcinoma, and non-Hodgkin’s lymphoma	Fine needle aspiration smears of cancer patients stained by MGG and H&E	Neutrophils internalized by tumor cells	All cases were associated with metastasis and high-grade tumors	[115]
Breast cancer	Patient tissue, H&E	Neutrophils engulfed by cancer cells	Not studied	[116]
Gastric adenocarcinomas	Patient tissue, H&E EM, TUNEL	Neutrophils engulfed by tumor cells	Not studied	[12,16,117]
Giant cell lung carcinoma	Patient tissue, H&E	Neutrophils engulfed by tumor cells	Not studied	[13]
Gallbladder carcinoma	Patient tissue, H&E	Neutrophils engulfed by tumor cells	Not studied	[14]
Pleomorphic xanthoastrocytomas	Patient tissue, H&E	Neutrophils engulfed by tumor cells	Not studied	[17]
Invasive micropapillary carcinoma (breast)	Patient tissue, H&E	Neutrophils engulfed by tumor cells	Not studied	[15]
Buccal mucosa squamous cell carcinoma	In vitro coculture of differentially labeled tumor cells and neutrophils; patient tissue, IHC	Neutrophils engulfed by tumor cells	Correlates with worse prognosis and survival	[110]
Spindle cell squamous cell carcinoma	Patient tissue H&E, IHC	Neutrophils engulfed by tumor cells	Not studied	[111]

## Abbreviations

TANs, tumor-associated neutrophils; LAP, LC3-associated phagocytosis; VPS34, vacuolar protein sorting 34; RUBCN, RUN domain and cysteine-rich domain containing Beclin-1-interacting protein; UVRAG, UV radiation resistance-associated gene protein; GMPs, granulocyte–monocyte progenitors; CD66b, Cluster of Differentiation 66b; STAT3, signal transducer and activator of transcription 3; G-CSFR, granulocyte colony-stimulating factor receptor; G-CSF, granulocyte colony-stimulating factor; IL-17, interleukin 17; IL-23, interleukin 23; CD11b, cluster of differentiation molecule 11B; CD16, cluster of differentiation 16; CXCL12, C-X-C motif chemokine 12; CXCR4, C-X-C chemokine receptor type 4; CXCL1, C-X-C motif chemokine ligand 1; CXCL2, C-X-C motif chemokine ligand 2; CXCL5, C-X-C motif chemokine ligand 5; CXCR2, C-X-C motif chemokine receptor 2; ROS, reactive oxygen species; RNS, reactive nitrogen species; 8-oxodG, 7-hydro-8-oxo-2′deoxyguanosine; miR-23a, microRNA-23a; miR-155, microRNA-155; MMP-9, matrix metalloproteinase 9; IRS-1, insulin receptor substrate-1; K-RAS, Kirsten rat sarcoma viral oncogene homologue; NETs, neutrophil extracellular traps; PTEN, phosphatase and tensin homolog; PD-L1, programmed death-ligand 1; CCL17, C-C motif chemokine ligand 17; T-regs, regulatory T cells; TRAIL, tumor necrosis factor-related apoptosis-inducing ligand; CD8, cluster of differentiation 8; IL-18, interleukin 18; NK, natural killer; HGFR, hepatocyte growth factor receptor; ADCC, antibody-dependent cell-mediated cytotoxicity; MPO, myeloperoxidase; H_2_O_2_, hydrogen peroxide; TME, tumor microenvironment; CAR, chimeric antigen receptor; LC3, microtubule-associated protein 1A/1B-light chain; Dectin-1, dendritic cell-associated C-type lectin-1; RhoA, Ras homolog family member A; ROCK, Rho-associated protein kinase; ATG5, autophagy-related 5; ATG7, autophagy-related 7; TLR, toll-like receptor; RGDS, tetrapeptide Arg-Gly-Asp-Ser; TIM-4, T cell immunoglobulin and mucin domain containing 4; PI3KC3, class III phosphatidylinositol 3-kinase complex; VPS15, phosphoinositide 3-kinase regulatory subunit 4; BECN1, Beclin-1; PI(3)P, phosphatidylinositol-3-phosphate; RAC1, Ras-related C3 botulinum toxin substrate 1; NOX2, NADPH oxidase 2; ATG4, autophagy-related 4; ATG3, autophagy-related 3; ATG12, autophagy-related 12; ATG16L1, autophagy-related 16 like 1; TAMs, tumor-associated macrophages; STING, stimulator of interferon genes; IFN, interferon; CD4, cluster of differentiation 4; ATG14, autophagy-related 14; ULK1, Unc-51-like autophagy activating kinase 1; FIP200, focal adhesion kinase family-interacting protein of 200 kDa; CXCL10, C-X-C motif chemokine ligand 10; CCL5, C-C motif chemokine ligand 5; STAT1, signal transducer and activator of transcription 1; IRF7, interferon regulatory factor 7; AML, acute myeloid leukemia.
